# Supplementary material for: Molecular choreography of primer synthesis by the eukaryotic Pol α-primase
Source: Nat Commun. 2023 Jun 21;14:3697. doi: 10.1038/s41467-023-39441-1 (PMC10284912; doi:10.1038/s41467-023-39441-1)
Supplement: Supplementary file 1 — Supplementary information [file 41467_2023_39441_MOESM1_ESM.pdf]

Supplementary information for

Molecular choreography of primer synthesis by the eukaryotic Pol α

By Yuan et al.

This document contains

3 Supplementary Tables

11 Supplementary Figures

Supplementary Table 1. Cryo-EM data collection and refinement statistics

**Data Collection**	**Apo Pol α (Conf I)**	**Apo Pol α (Conf II)**	**Pol α–T**	**Pol α–T/P8**	**Pol α–T/P10**	**Pol α–T/P15**
EM equipment	FEI Titan Krios	FEI Titan Krios	FEI Titan Krios	FEI Titan Krios	FEI Titan Krios	FEI Titan Krios
Voltage (kV)	300	300	300	300	300	300
Detector	Gatan K3	Gatan K3	Gatan K3	Gatan K3	Gatan K3	Gatan K3
Pixel size (Å)	0.828	0.828	0.828	0.828	0.828	0.828
Electron dose (e^-/Å^2)	50	50	50	50	50	50
Defocus range (-µm)	1.0-1.5	1.0-1.5	1.0-1.5	1.0-1.5	1.0-1.5	1.0-1.5
**Reconstruction**						
Software	RELION 3.1	RELION 3.1	RELION 3.1	RELION 3.1	RELION 3.1	RELION 3.1
Particles number	111,513	136,294	176,443	186,967	164,567	542,937
Resolution (Å)	3.7	3.8	5.6	4.8	4.5	3.5
Map sharpening B-factor (Å^2)	-145	-150	-230	-197	-184	-122
**Model composition**						
Peptide chains	4	4	4	4	4	4
Protein residues	2287	2287	2287	2287	2287	2287
**R.m.s. deviations**						
Bonds length (Å)	0.011	0.006	0.013	0.013	0.009	0.009
Bonds Angle (°)	1.088	1.126	1.398	1.501	1.149	1.189
**Ramachandran plot**						
Preferred (%)	98.54	97.08	97.75	97.34	96.38	97.62
Allowed (%)	1.42	2.83	2.25	2.61	3.62	2.38
Outlier (%)	0.04	0.09	0.05	0.05	0.00	0.30
**Validation**						
Molprobity score	1.51 (95%)	1.66 (90%)	1.12 (93%)	1.39 (97%)	1.75(87%)	1.58(92%)
Clash score	9.81 (72%)	9.47(74%)	2.78 (98%)	5.03(94%)	9.77(73%)	9.34(74%)
Rotamer outliers (%)	0.24	0.21	0.67	0.65	0.63	0.15

Supplementary Table 2. Oligonucleotides used in this cryo-EM study

Oligonucleotides	Sequence
**Template**	TTTTTTTTTTTTTTTTTTTTTTTTTTTTTTTTTTTTCTTTTTTTCGCTGCCCGCCTTTTTT
**Primer P6**	AGGCGG
**Primer P7**	AGGCGGG
**Primer P8**	AGGCGGGC
**Primer P9**	AGGCGGGCA
**Primer P10**	AGGCGGGCAG
**Primer P11**	AGGCGGGCAGC
**Primer P15**	AGGCGGGCAGCGAAA

All sequences are listed in the order from 5' to 3'. Ribonucleotides are underlined.

Supplementary Table 3. Correlation coefficient between model and map. CC values were calculated by Phenix validation. Chain ID 1 refers to Pol1, A to Pri1, B to Pri2, C to Pol12, T to DNA template, and P to RNA or DNA/RNA primer.

EM map I (Pol α apo I, 3.7 Å)

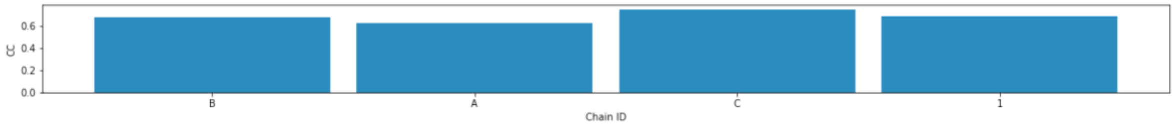

EM map II (Pol α apo II, 3.8 Å)

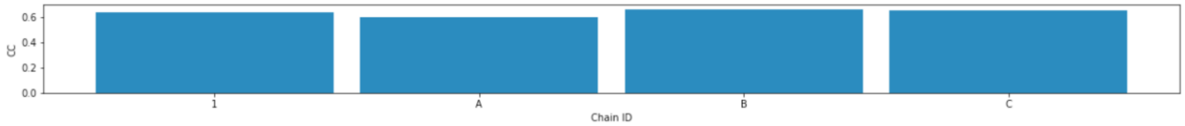

EM map III (Pol α-T, 5.6 Å)

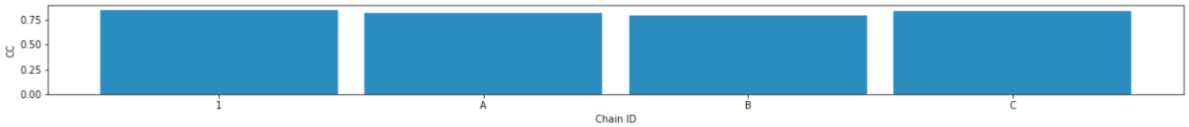

EM map IV (Pol α-T/P8, 4.6 Å)

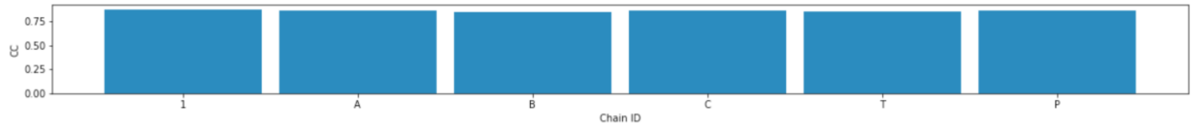

EM map V (Pol α-T/P10, 4.5 Å)

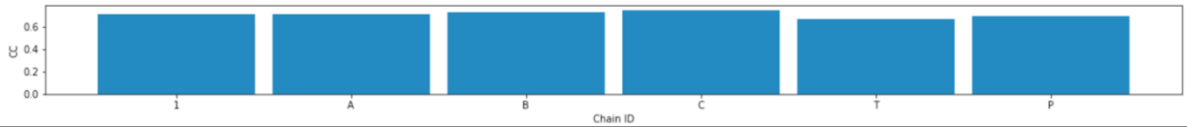

EM map VI (Pol α-T/P15, 3.5 Å)

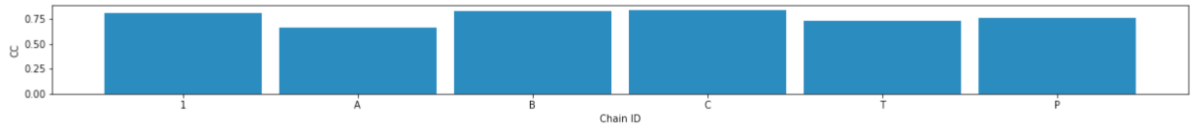

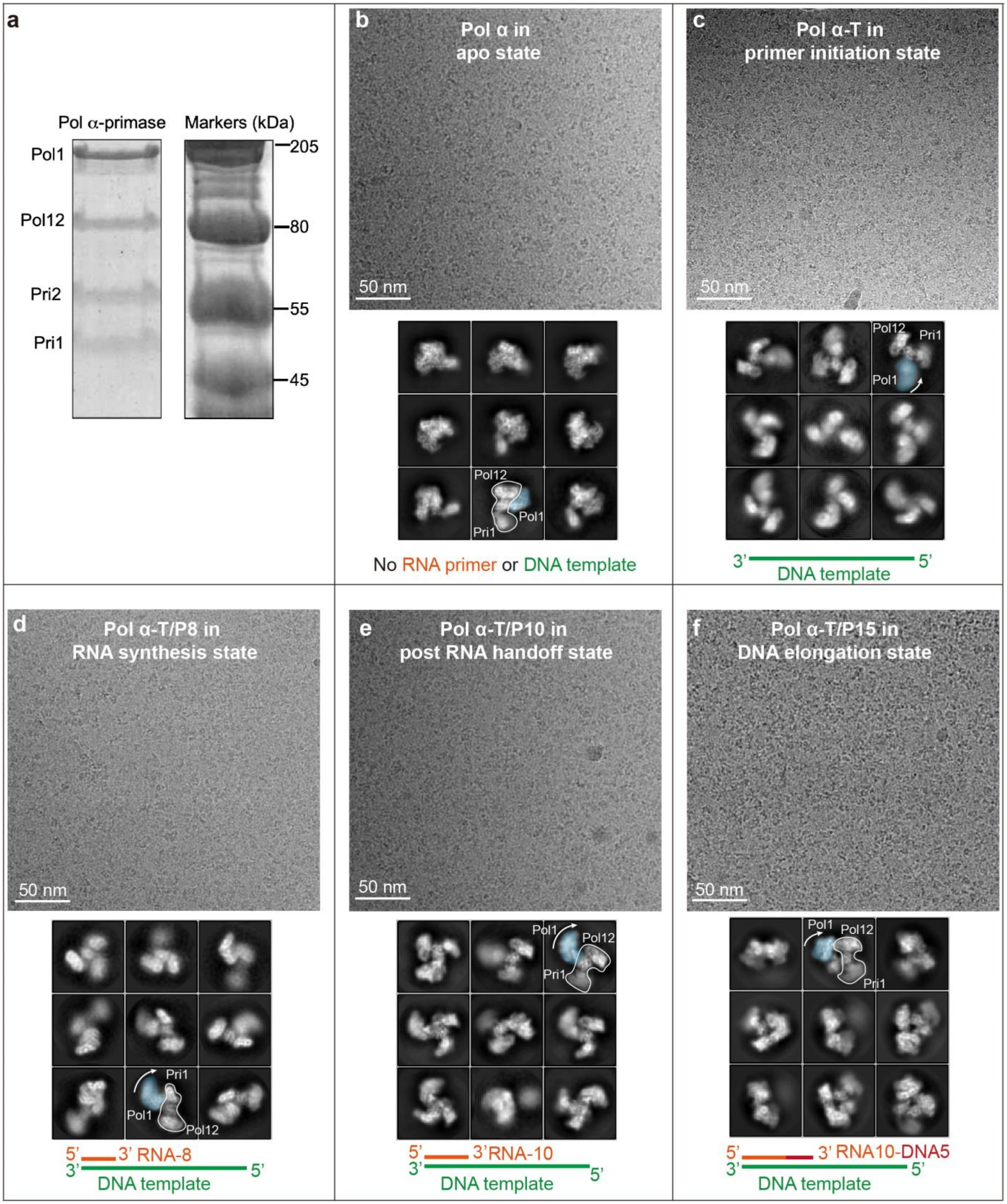

Supplementary Figure 1. Pol α complex sample characterization and cryo-EM. a) Coomassie blue stained SDS-PAGE gel of the Pol α-primase preparation, showing the presence of all four subunits. Source data are provided as a Source Data file. **b-f**) Upper rows show typical raw micrographs of Pol α-primase complexed with various T/P substrates as sketched in the bottom row of each panel. The raw micrographs were selected from 10,529, 3,357, 14,691, 13,433, and 17,525 micrographs, respectively. The middle rows show selected 2D class averages in different views.

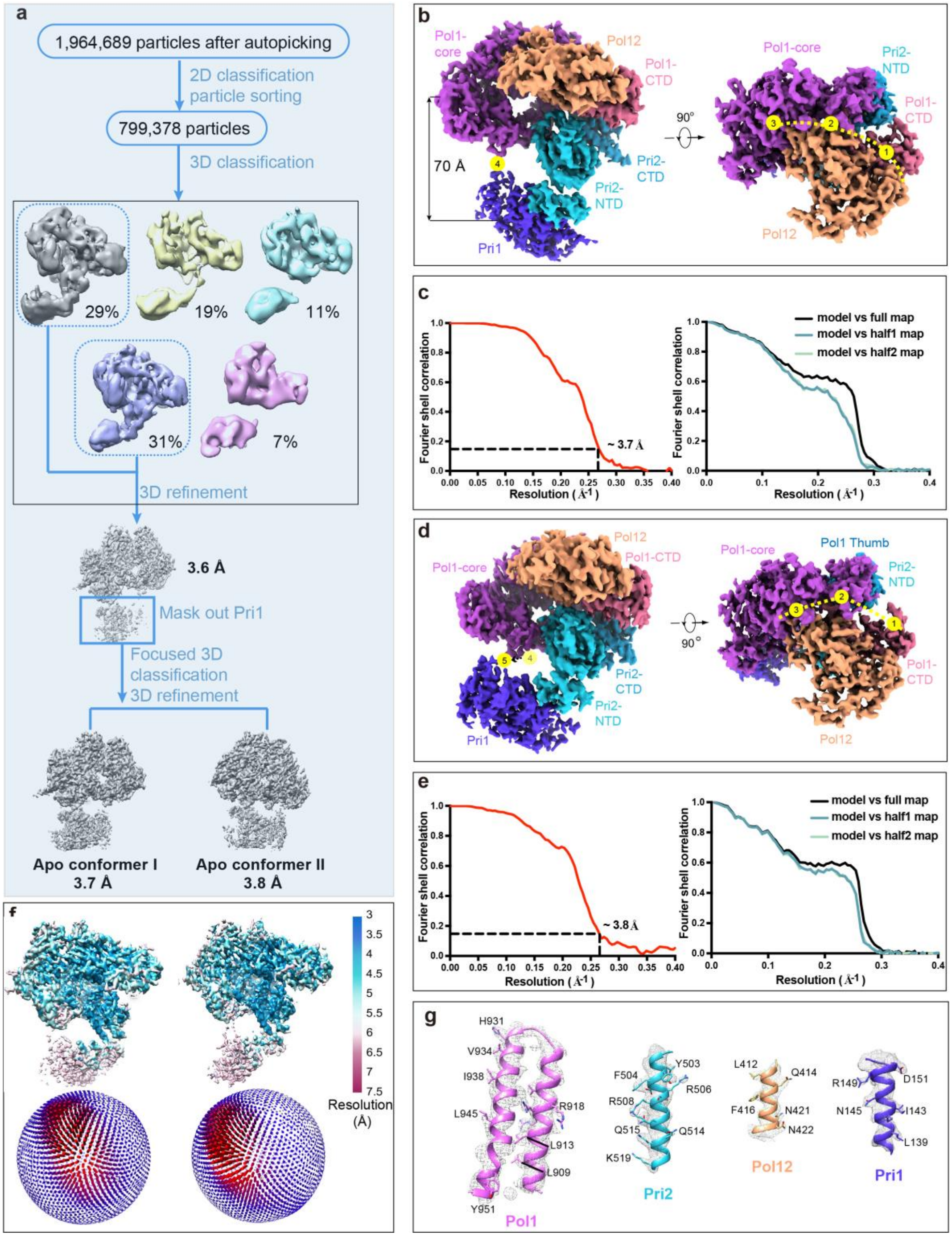

Supplementary Figure 2. Cryo-EM of the Pol α-primase in apo state conformers I and II. a) 3D classification procedure used to derive the two 3D EM maps. The first and forth classes were combined for further refinement, leading to the 3.7-Å map (conformer I) and the 3.8-Å map (conformer II). The other three classes were discarded. **b**) Surface-rendered EM map in state I in a front and a top view. **c**) Resolution estimation by gold-standard Fourier shell correlation at 0.143 (left) and model vs maps (right). Source data are provided as a Source Data file. **d**) The conformer II EM map in a front and a top view. **e**) Gold-standard Fourier shell correlation estimation of the EM map resolution at 0.143 (left) and model vs maps (right). **f**) Color-coded local resolution maps of the Apo Pol α-primase in conformers I and II. Lower panels are euler angle distribution of all particles included in the final 3D reconstructions of conformers I and II. **g**) Model-map fitting of the apo Pol α-primase in four selected regions as labeled.

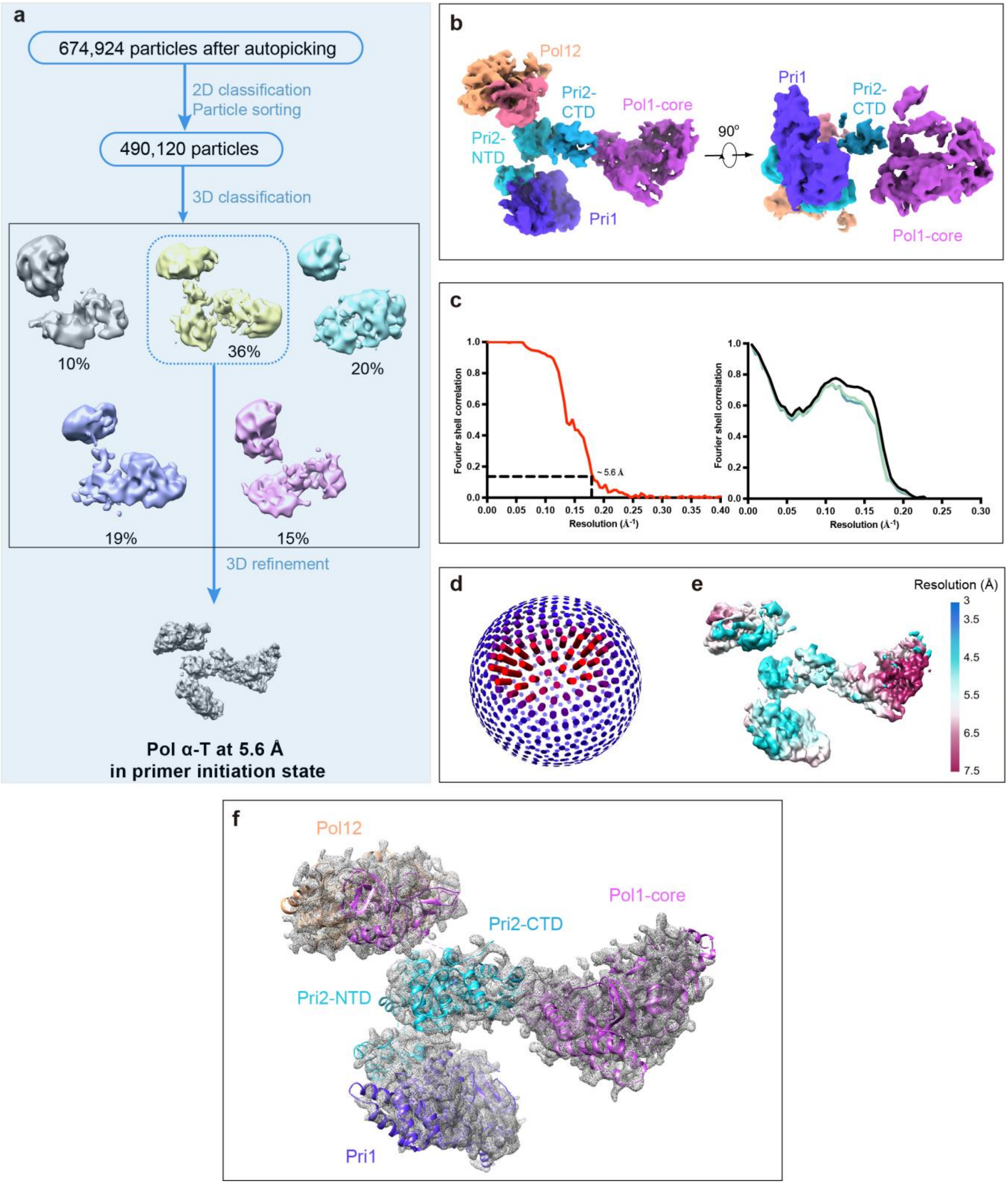

Supplementary Figure 3. Cryo-EM of the Pol α–T complex (primer initiation state). a) 3D classification procedure used to derive the EM map. The second class was selected for further refinement, leading to the final map at 5.6 Å overall resolution. The other four classes were discarded. **b**) Surface-rendered EM map in a front and top views. **c**) Gold standard Fourier shell correlation estimation at the 0.143 correlation threshold (left) and the correlations between model and maps (right). Source data are provided as a Source Data file. **d**) Euler angle distribution of all particles included in the final 3D reconstruction. **e**) Color-coded local resolution estimation of the EM map. **f**) Atomic model in cartoons superimposed on EM map rendered in gray meshes.

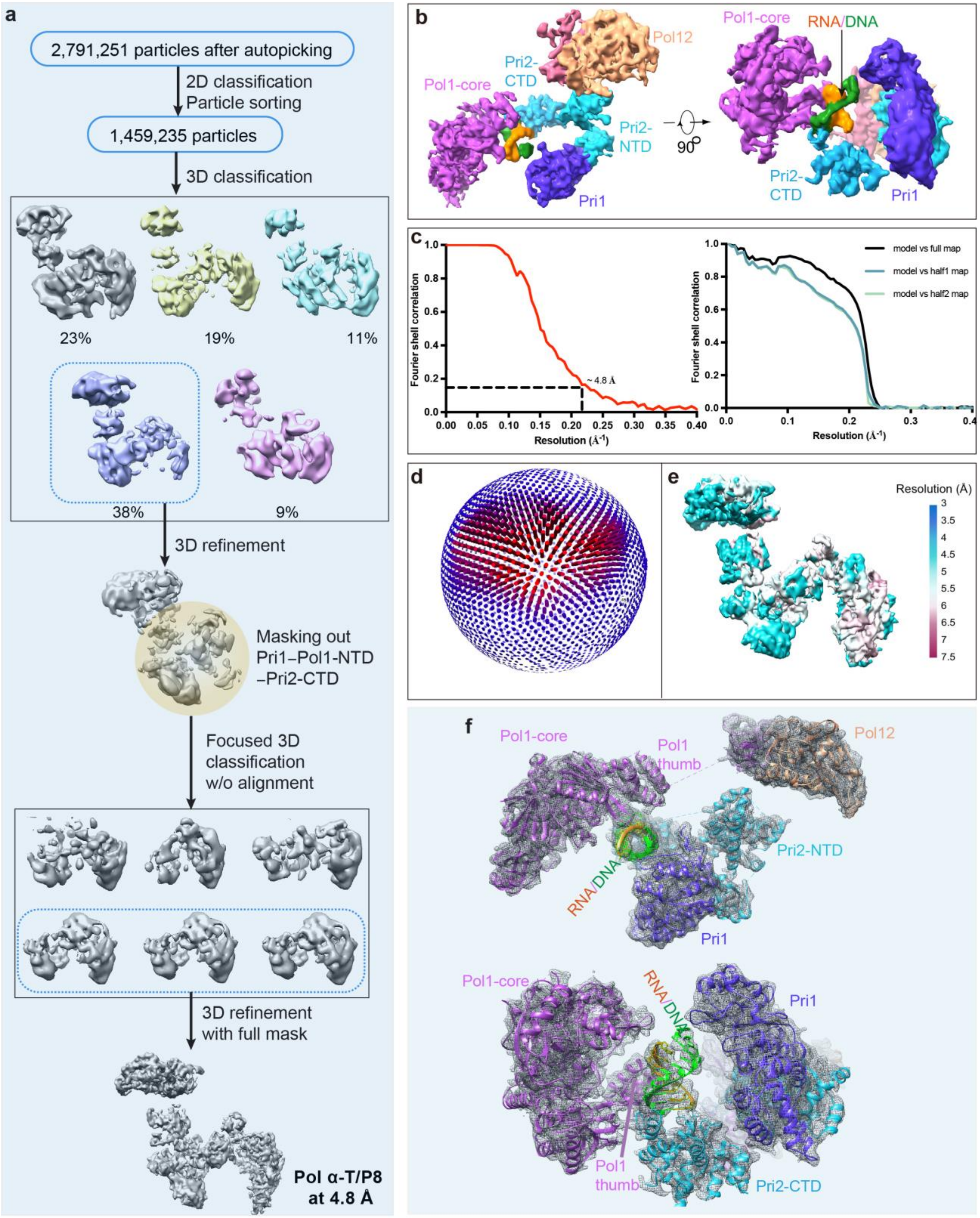

Supplementary Figure 4. Cryo-EM of the Pol α–T/P8 complex (RNA synthesis state). a) 3D classification procedure used to derive the EM map. The fourth class was selected for further refinement, leading to the final map at 4.8 Å overall resolution. The other four classes were discarded. **b**) Surface-rendered EM map in a front and a top view. **c**) Resolution estimation by gold standard Fourier shell correlation at the 0.143 threshold (left) and correlations between model and maps (right). Source data are provided as a Source Data file. **d**) Euler angle distribution of particles included in the final 3D reconstruction. **e**) Color-coded local resolution estimation of the EM map. **f**) Atomic model in cartoons superimposed on the EM map rendered in gray meshes.

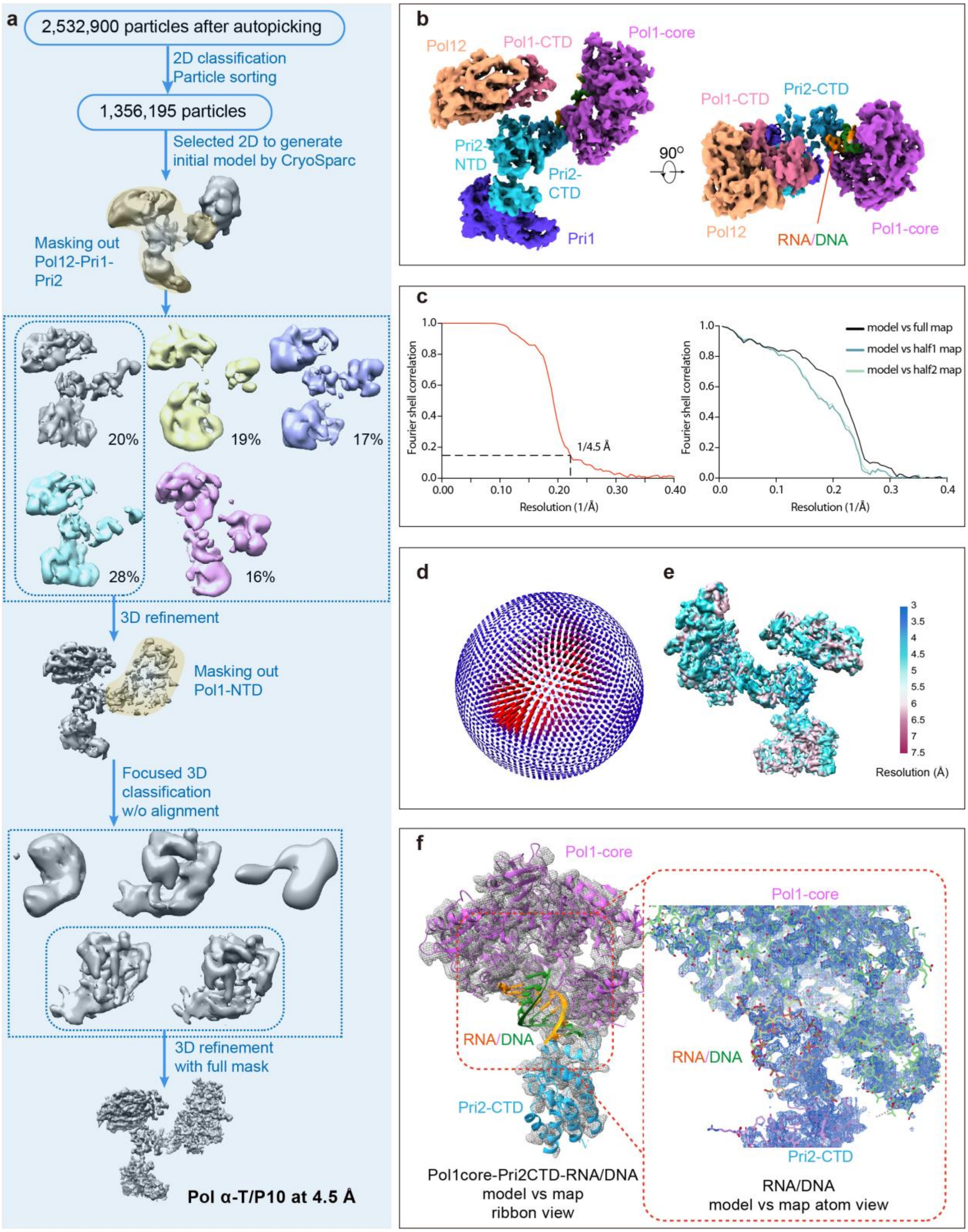

Supplementary Figure 5. Cryo-EM of the Pol α–T/P10 complex (RNA hand-off state). a) 3D classification procedure used to derive the EM map. The first and fourth classes were combined for further refinement, leading to the final map at 4.5 Å overall resolution. The other three classes were discarded. **b**) The surface-rendered map (state V) in a front and the top view. **c**) Resolution estimation of the EM map by gold standard Fourier shell correlation at the correlation threshold of 0.143 (left) and correlations between the model and maps (right). Source data are provided as a Source Data file. **d**) Euler angle distribution of particles used in the final reconstruction. **e**) Colored-coded local resolution estimation of the EM map. **f**) Fitting of the model and EM map with a zoomed view in the T/P region.

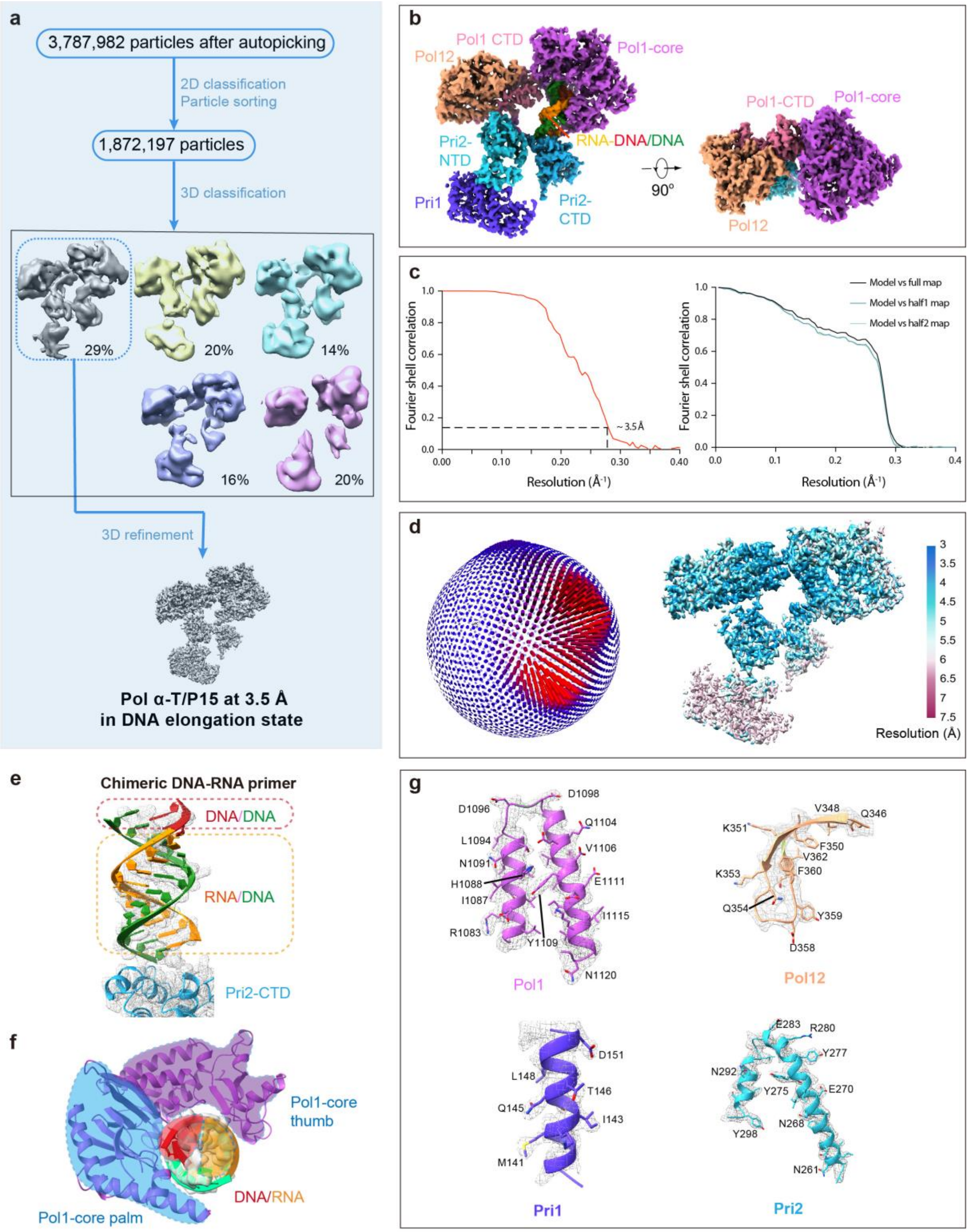

Supplementary Figure 6. Cryo-EM of the Pol α–T/P15 complex (DNA elongation state). a) 3D classification procedure used to derive the 3D EM map. The first 3D class was selected for further refinement leading to the final map at 3.5 Å resolution. The other four classes were discarded. **b**) The EM map in a front and the top view. **c**) Gold standard Fourier shell correlation estimation (left) and correlations between the model and the maps (right). Source data are provided as a Source Data file. **d**) Euler angle distribution of raw particles included in the final reconstruction (left) and color-coded local resolution map of the EM map (right). **e**) Fitting of the T/P15 model in the EM map. **f**) The Pol1 thumb engages the primer side while palm engages the template side of the T/P15. **g**) Model-density fitting in selected regions of Pol1, Pol12, Pri2, and Pri1.

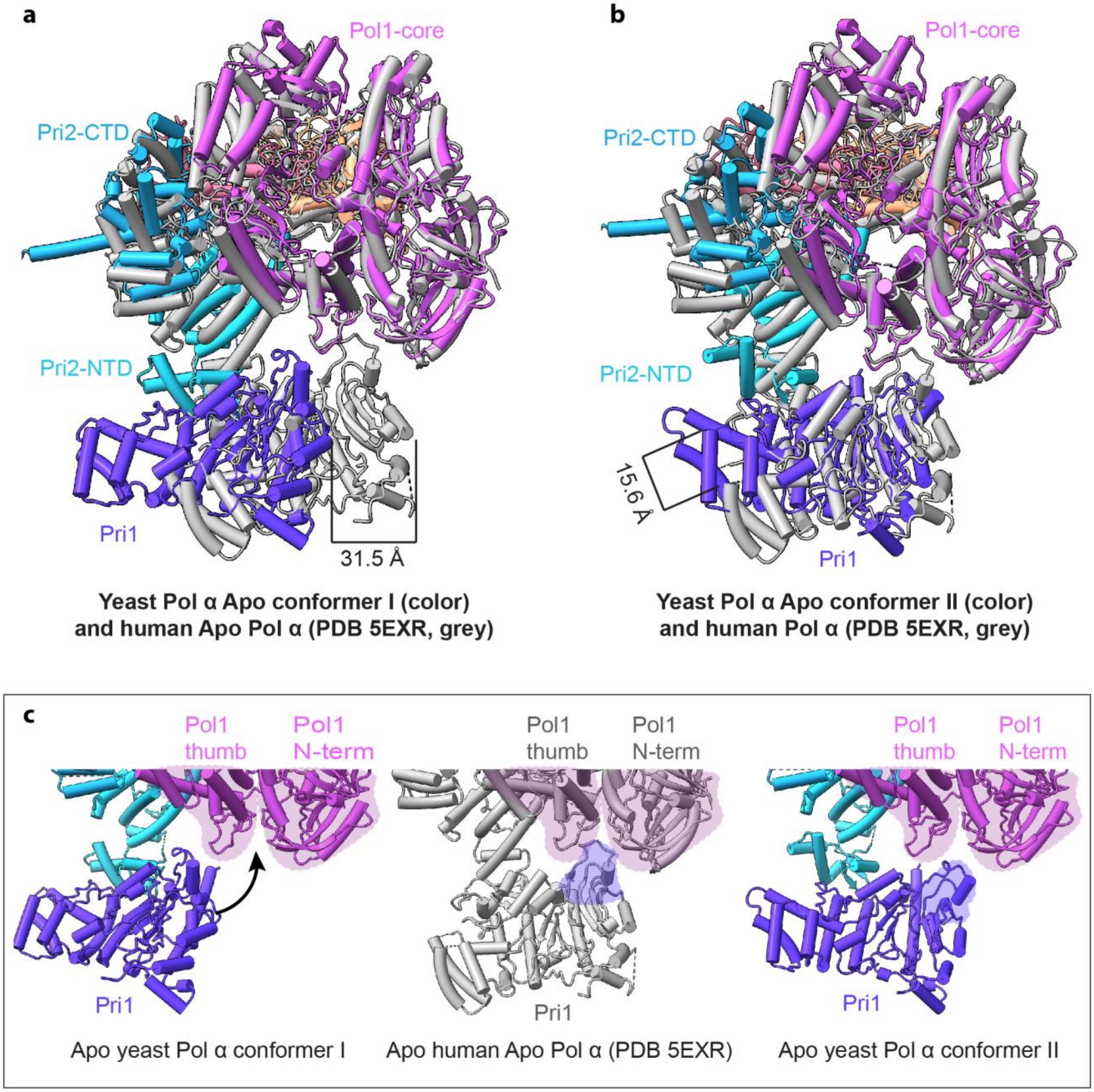

Supplementary Figure 7. Comparison of the yeast and human Pol α-primase both in the apo state. a-b) Superimposition of the yeast Pol α-primase in conformer I (a) and conformer II (b) with the human Pol α-primase crystal structure (PDB ID 5EXR). **c**) Side-by-side comparison of the Pri1 interaction with the Pol1-core thumb and N-term in the yeast Pol α-primase in conformers I and II and the human Pol α-primase.

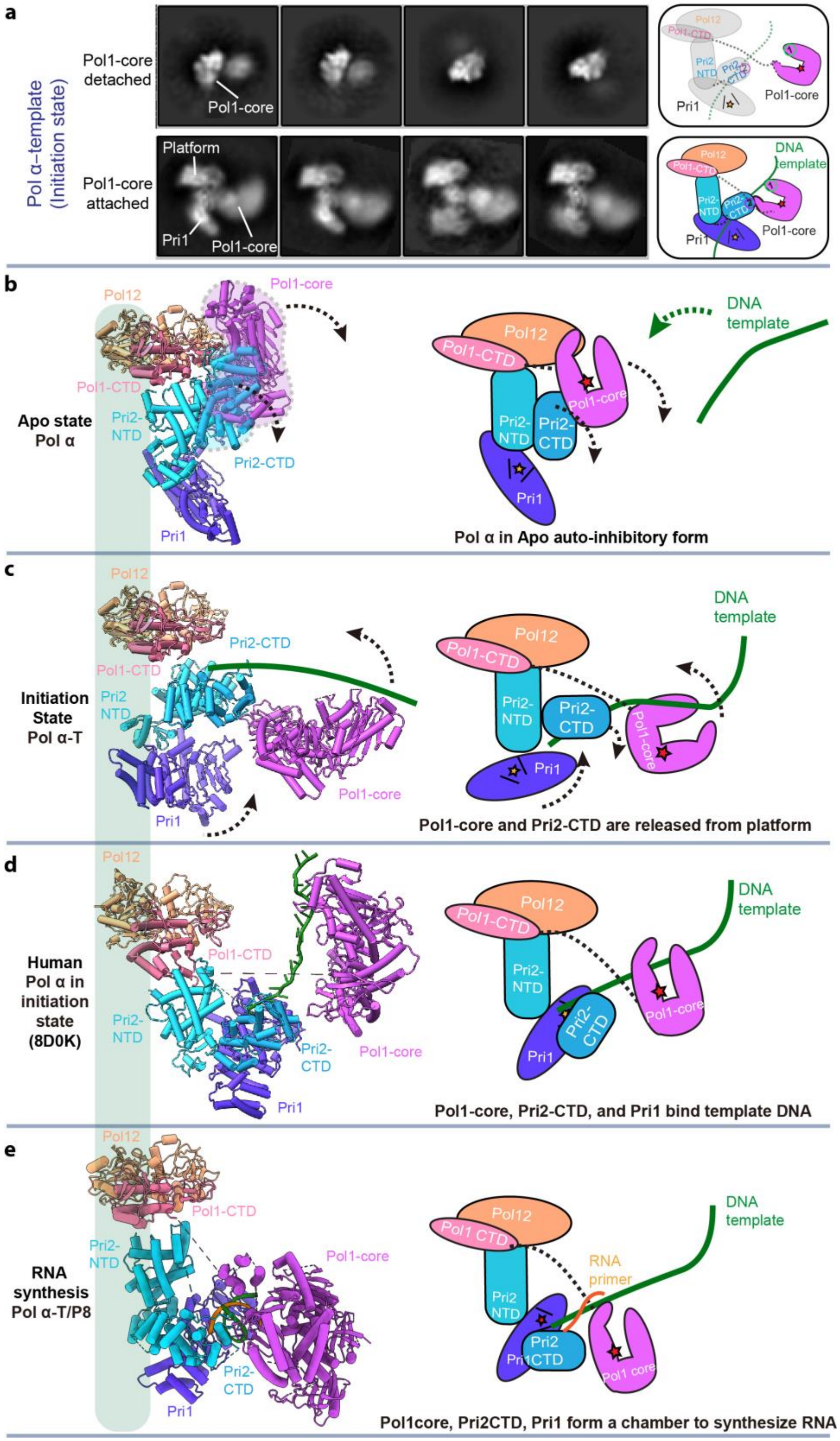

Supplementary Figure 8. **Comparison of the yeast Pol α–T with the human structure stabilized by CST.** **a**) 2D classification of the yeast Pol α–T complex. Upper row shows particles with fully released Pol1-core from Pol12, and lower row is partly released with attachment to Pol12 platform via Pri2-CTD and the linker of Pol1-NTD and Pol1-CTD. **b-e**) Comparison of the Pol1-core positions in the yeast Pol α apo (b), in the primer initiation state (c), in the human Pol α–T in the primer initiation state (d, PDB ID 8D0K), and in the yeast Pol α in the RNA synthesis state (e).

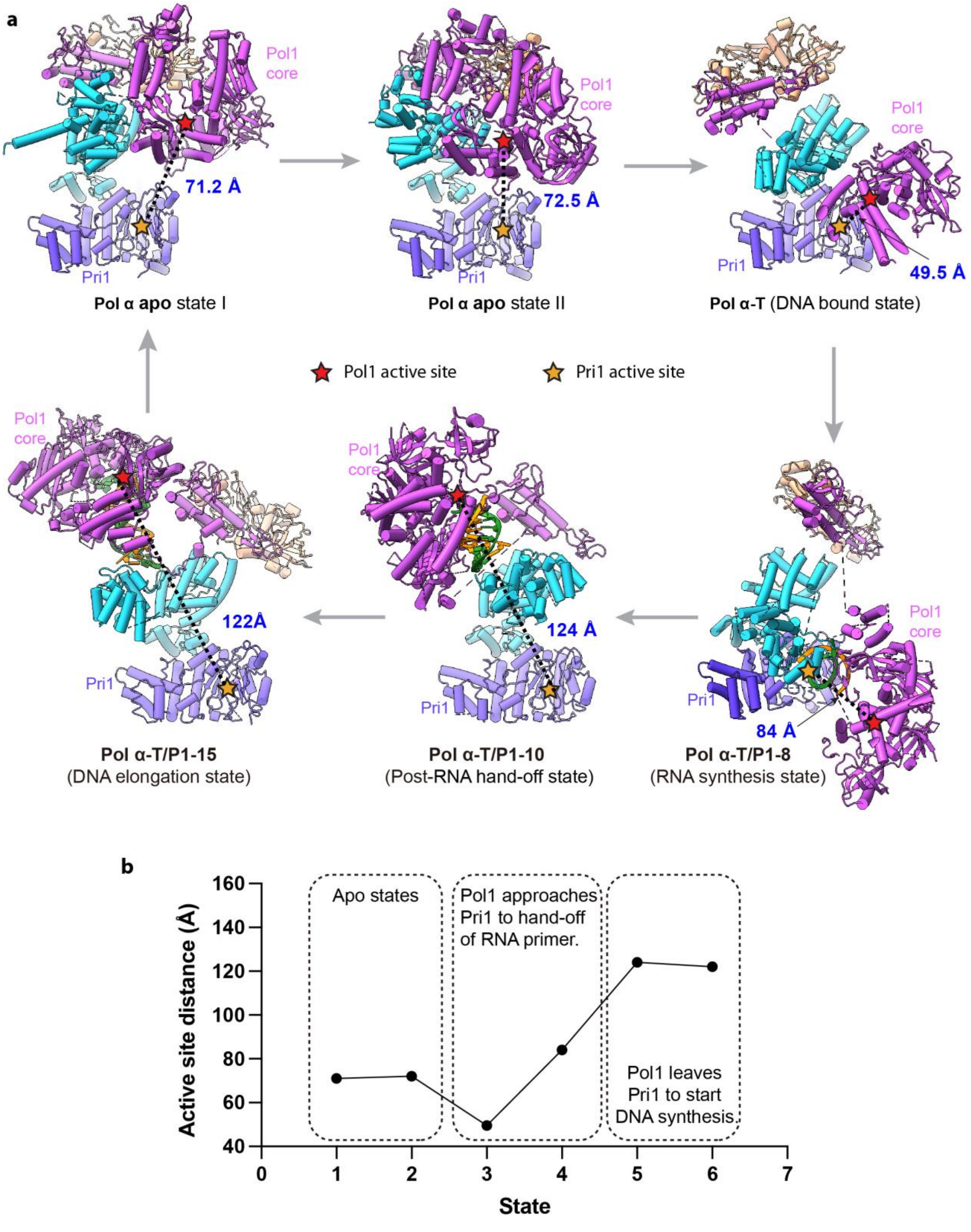

Supplementary Figure 9. The distance between the primase and the polymerase active sties varies significantly as Pol α goes through the RNA/DNA primer synthesis process. a) In each panel the dashed line shows the distance between the Pol1 (red asterisk) and Pri1 active site (yellow asterisk). The distance is measured between Pri1 K326 and Pol1 R917, the two positively charged amino acids in the catalytic pockets of Pri1 and Pol1, respectively. **b**) A plot of the distances between the Pol1 and Pri1 active sites in the six captured states. Source data are provided as a Source Data file.

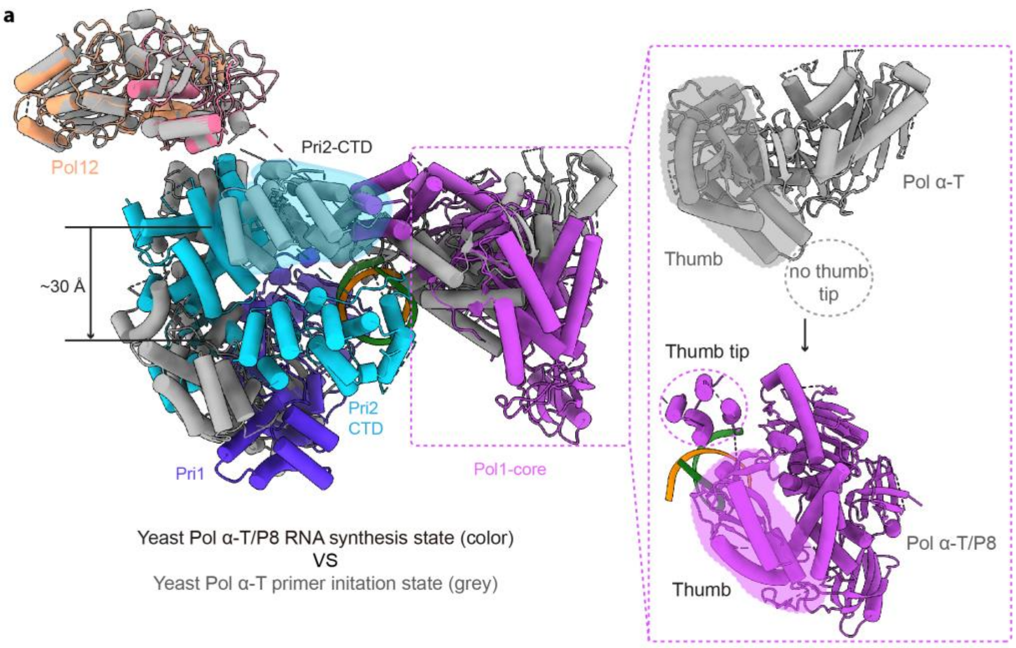

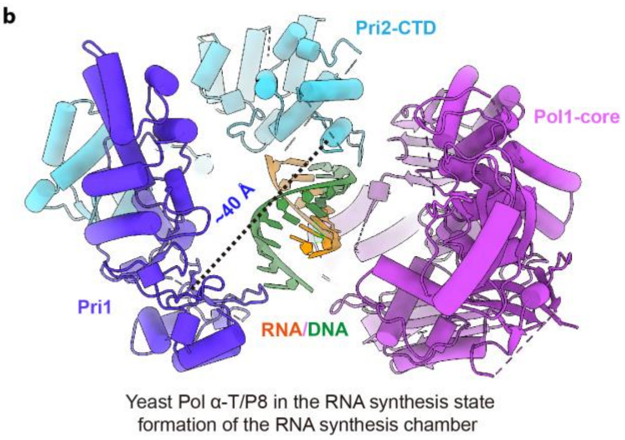

Supplementary Figure 10. Overall view of Pol α in the primer initiation and RNA synthesis states. a) Superimposition of the primer initiation state (gray cartoon) and the RNA synthesis state (color cartoon), highlighting a ~30 Å movement of Pri2-CTD between the two states. The enlarged view to the right shows the stabilized tip of the Pol1 thumb domain during RNA synthesis. The tip is disordered in the initiation state. **b**) Structure of the Pol α in the RNA synthesis state. The dashed line shows the presence of a large chamber ~40 Å across encircled by Pri1, Pri2-CTD, and Pol1-core.

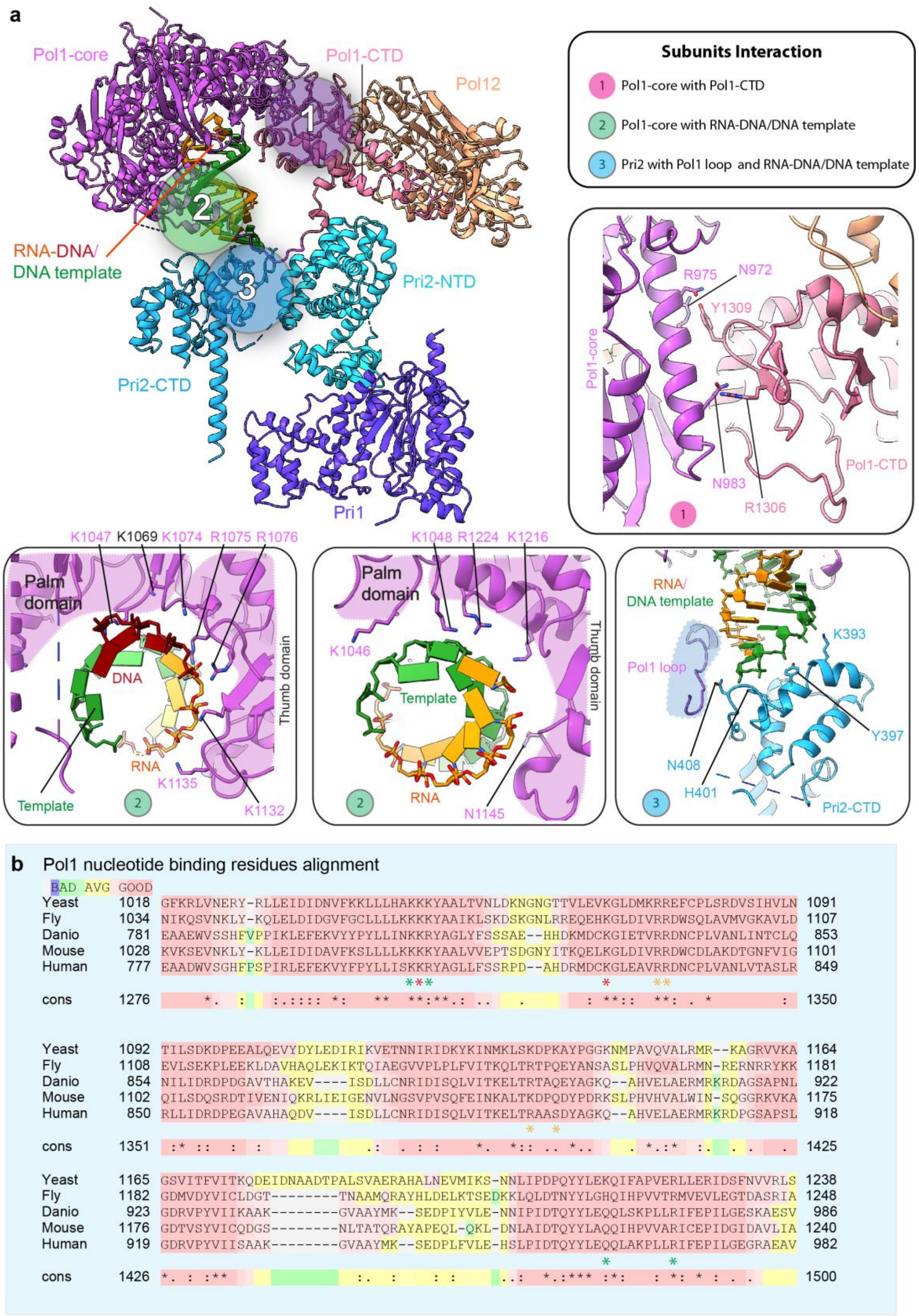

Supplementary Figure 11. Key Interactions in the Pol α–T/P15 complex (DNA elongation state). a) Overall structure of the yeast Pol α-primase in the DNA elongation state. The numbered circles mark the contact regions between different domains that are enlarged in separate panels: panel 1 is between Pol1-core and Pol1-CTD; 2 is between Pol1-core and T/P15; 3 is between Pri2, the Pol1 loop, and T/P15. **b**) Alignment of the Pol1-core nucleotide binding residues in the yeast, fly, fish, mouse, and human. Red asterisks indicate conserved residues that contact the T/P15.
