# Supplementary material for: Molecular choreography of primer synthesis by the eukaryotic Pol α-primase
Source: Nat Commun. 2023 Jun 21;14:3697. doi: 10.1038/s41467-023-39441-1 (PMC10284912; doi:10.1038/s41467-023-39441-1)
Supplement: Supplementary file 3 — Description of Additional Supplementary Files [file 41467_2023_39441_MOESM3_ESM.pdf]

Description of Additional Supplementary Files

File Name: Supplementary Movie 1
Description: Morphing of the yeast Pol α-primase complex as it goes through the five major states of primer synthesis, starting with the two conformations in apo state when no template DNA is bound, to the primer initiation state in which a 60-nt DNA template is present, to the RNA primer synthesis state bound to a T/P8, to the RNA primer hand-off state bound to a T/P10, and finally to the DNA elongation state bound to a T/P15.
