# Supplementary material for: Molecular choreography of primer synthesis by the eukaryotic Pol α-primase
Source: Nat Commun. 2023 Jun 21;14:3697. doi: 10.1038/s41467-023-39441-1 (PMC10284912; doi:10.1038/s41467-023-39441-1)
Supplement: Supplementary file 5 — Reporting Summary [file 41467_2023_39441_MOESM5_ESM.pdf]

nature portfolio

Corresponding author(s): Huilin Li and Michael O'Donnell

Last updated by author(s): May 25, 2023

Reporting Summary

Nature Portfolio wishes to improve the reproducibility of the work that we publish. This form provides structure for consistency and transparency in reporting. For further information on Nature Portfolio policies, see our Editorial Policies and the Editorial Policy Checklist.

Statistics

For all statistical analyses, confirm that the following items are present in the figure legend, table legend, main text, or Methods section.

n/a	Confirmed	
☒	☐	The exact sample size (*n*) for each experimental group/condition, given as a discrete number and unit of measurement
☐	☒	A statement on whether measurements were taken from distinct samples or whether the same sample was measured repeatedly
☒	☐	The statistical test(s) used AND whether they are one- or two-sided<br>*Only common tests should be described solely by name; describe more complex techniques in the Methods section.*
☒	☐	A description of all covariates tested
☒	☐	A description of any assumptions or corrections, such as tests of normality and adjustment for multiple comparisons
☒	☐	A full description of the statistical parameters including central tendency (e.g. means) or other basic estimates (e.g. regression coefficient) AND variation (e.g. standard deviation) or associated estimates of uncertainty (e.g. confidence intervals)
☒	☐	For null hypothesis testing, the test statistic (e.g. F, t, r) with confidence intervals, effect sizes, degrees of freedom and P value noted<br>*Give P values as exact values whenever suitable.*
☒	☐	For Bayesian analysis, information on the choice of priors and Markov chain Monte Carlo settings
☒	☐	For hierarchical and complex designs, identification of the appropriate level for tests and full reporting of outcomes
☒	☐	Estimates of effect sizes (e.g. Cohen's *d*, Pearson's *r*), indicating how they were calculated

Our web collection on statistics for biologists contains articles on many of the points above.

Software and code

Policy information about availability of computer code

Data collection	SerialEM version 4.0.0 beta was used to collect the cryoEM micrographs.
Data analysis	cryoSPARC v3.2, ChimeraX 1.2.5, Coot (version 0.9.5), Phenix (version 1.19.2-4158-000), MolProbity (version 4.5), and Pymol (version 2.5.0).

For manuscripts utilizing custom algorithms or software that are central to the research but not yet described in published literature, software must be made available to editors and reviewers. We strongly encourage code deposition in a community repository (e.g. GitHub). See the Nature Portfolio guidelines for submitting code & software for further information.

Data

Policy information about availability of data

All manuscripts must include a data availability statement. This statement should provide the following information, where applicable:

- Accession codes, unique identifiers, or web links for publicly available datasets
- A description of any restrictions on data availability
- For clinical datasets or third party data, please ensure that the statement adheres to our policy

The PDB and EMDB accession codes of the six structures determined in the current work are:
Apo Pol alpha in conformation I: PDB: 8FOC [http://doi.org/10.2210/pdb8FOC/pdb]; EMDB: EMD-29345 [https://www.ebi.ac.uk/emdb/EMD-29345]
Apo Pol alpha in conformation II: PDB: 8FOD [http://doi.org/10.2210/pdb8FOD/pdb]; EMDB: EMD-29346 [https://www.ebi.ac.uk/emdb/EMD-29346]
Pol alpha bound to a DNA template: PDB: 8FOE [http://doi.org/10.2210/pdb8FOE/pdb]; EMDB: EMD-29347 [https://www.ebi.ac.uk/emdb/EMD-29347]

Pol alpha bound to a T/P8: PDB: 8FOH [http://doi.org/10.2210/pdb8FOH/pdb]; EMDB: EMD-29349 [https://www.ebi.ac.uk/emdb/EMD-29349]
Pol alpha bound to a T/P10: PDB: 8FOJ [http://doi.org/10.2210/pdb8FOJ/pdb]; EMDB: EMD-29351 [https://www.ebi.ac.uk/emdb/EMD-29351]
Pol alpha bound to a T/P15: PDB: 8FOK 8FOK [http://doi.org/10.2210/pdb8FOK/pdb]; EMDB: EMD-29352 [https://www.ebi.ac.uk/emdb/EMD-29352].
Additionally, this study used the following previously published structures:
PDB ID 3LGB [http://doi.org/10.2210/pdb3LBG/pdb]; PDB ID 4BPU [http://doi.org/10.2210/pdb4BPU/pdb]; PDB ID 3FLO [http://doi.org/10.2210/pdb3FLO/pdb]; PDB ID 4B08 [http://doi.org/10.2210/pdb4B08/pdb]; PDB ID 5EXR [http://doi.org/10.2210/pdb5EXR/pdb].

Human research participants

Policy information about studies involving human research participants and Sex and Gender in Research.

Reporting on sex and gender	N/A
Population characteristics	N/A
Recruitment	N/A
Ethics oversight	N/A

Note that full information on the approval of the study protocol must also be provided in the manuscript.

Field-specific reporting

Please select the one below that is the best fit for your research. If you are not sure, read the appropriate sections before making your selection.

☒ Life sciences ☐ Behavioural & social sciences ☐ Ecological, evolutionary & environmental sciences

For a reference copy of the document with all sections, see nature.com/documents/nr-reporting-summary-flat.pdf

Life sciences study design

All studies must disclose on these points even when the disclosure is negative.

Sample size	We collected a dataset of 10,519 raw movie micrographs for Pol α alone in the apo form, a dataset of 3357 raw movie micrographs for Pol α-template (T) complex, a dataset of 14,691 micrographs for the Pol α–T/P8 complex, one dataset of 13,433 micrographs for the Pol α–T/P10 complex, and one dataset of 17,525 micrographs for the Pol α–T/P15 complex. Sample size was not predetermined. The sample sizes were deemed sufficient either the targeted resolution has achieved or when the resolution of the derived 3D EM map no longer improved.
Data exclusions	"Bad" raw particle images that did not produce 2D class averages or 3D class maps with defined features were excluded after 2D and 3D classifications. This criteria is empirical but is a standard image processing practice in the cryo-EM community.
Replication	Reproducibility resides in the large number of particles used to derive at the final 3D maps or 2D averages. The reliability and the resolution is measured by gold-standard Fourier shell correlation. Replication efforts with multiple refinement runs yielded was successful, yielding similar 3D maps. The six datasets were collected in different EM sessions, and the EM grids were made in different time and independently.
Randomization	The raw particles were automatically selected by blob picking of the computer program (cryoSPARC v3.2). Randomization is irrelevant to this study.
Blinding	The investigators were not blinded to the specific data points during data collection and analysis, because visual inspection is necessary to ascertain the data quality.

Reporting for specific materials, systems and methods

We require information from authors about some types of materials, experimental systems and methods used in many studies. Here, indicate whether each material, system or method listed is relevant to your study. If you are not sure if a list item applies to your research, read the appropriate section before selecting a response.

Materials & experimental systems

n/a	Involved in the study
☒	☐ Antibodies
☒	☐ Eukaryotic cell lines
☒	☐ Palaeontology and archaeology
☒	☐ Animals and other organisms
☒	☐ Clinical data
☒	☐ Dual use research of concern

Methods

n/a	Involved in the study
☒	☐ ChIP-seq
☒	☐ Flow cytometry
☒	☐ MRI-based neuroimaging
